# Why carers use adult day respite: a mixed method case study

**DOI:** 10.1186/1472-6963-14-245

**Published:** 2014-06-06

**Authors:** Christine M Stirling, Corinna A Dwan, Angela R McKenzie

**Affiliations:** 1 School of Health Sciences, University of Tasmania, Private Bag 135, Hobart, Tasmania 7000, Australia; 2 School of Health Sciences, Wicking Dementia Research and Education Centre, University of Tasmania, Private Bag 135, Hobart, Tasmania 7000, Australia; 3 Glenview Community Services, 2-10 Windsor St, Glenorchy, Tasmania 7010, Australia

**Keywords:** Ageing, Carer, Day respite, Maslow’s hierarchy of need, Psychological contract

## Abstract

**Background:**

We need to improve our understanding of the complex interactions between family carers’ emotional relationships with care-recipients and carers use of support services. This study assessed carer’s expectations and perceptions of adult day respite services and their commitment to using services.

**Methods:**

A mixed-method case study approach was used with psychological contract providing a conceptual framework. Data collection was situated within an organisational case study, and the total population of carers from the organisation’s day respite service were approached. Fifty respondents provided quantitative and qualitative data through an interview survey. The conceptual framework was expanded to include Maslow’s hierarchy of needs during analysis.

**Results:**

Carers prioritised benefits for and experiences of care-recipients when making day respite decisions. Respondents had high levels of trust in the service and perceived that the major benefits for care-recipients were around social interaction and meaningful activity with resultant improved well-being. Carers wanted day respite experiences to include all levels of Maslow’s hierarchy of needs from the provision of physiological care and safety through to the higher levels of belongingness, love and esteem.

**Conclusion:**

The study suggests carers need to trust that care-recipients will have quality experiences at day respite. This study was intended as a preliminary stage for further research and while not generalizable it does highlight key considerations in carers’ use of day respite services.

## Background

Respite care is considered a key support service for carers of the elderly, with the ascribed benefits including lower carer strain, and advantages for the care-recipient including delayed institutionalization [1]. Nevertheless these services remain under-utilized by carers [2] and the dynamics of carer and care-recipient interactions in relation to respite services are poorly understood and under theorized. A key aspect of care arrangements, which has been overlooked, is the role of carer trust and the relationship between ‘trust’ and respite usage. Literature acknowledges the strength of the carer/care-recipient dyad [3] and yet respite services are framed in terms of a ‘rest for carers’ [4]. This paper reports a study investigating carer expectations and perceptions of the benefits of using day respite and carers’ levels of trust and commitment in using these services to shed light on this under-researched area.

### The evidence for respite

Australian policy makers are looking for solutions to the anticipated surge in care costs and the predicted shortage of 250,000 residential aged care places by 2050 [5]. It is expected that these projected costs and placement shortages can be minimised if some demand is shifted from residential aged care facilities to community services.

Family members provide the bulk of care in the community setting [2] but many consequently suffer negative psychological, physical, financial, and social impacts [6-8]. Numerous studies have shown that carers experience high levels of strain resulting in poor health outcomes and lower quality of life for carers, and earlier institutionalization for care-recipients [9]. Policy makers have focused on respite care to help relieve the burden of caregiving and to facilitate ageing in place [10,11]. In spite of this 66% of carers in Australia do not use the service with a key reason being carers’ belief that respite will have negative outcomes for care recipients [12].

There are three main forms of respite care for the elderly – in-home respite, day respite or longer institutional based care, all aimed at decreasing carer burden and with the potential to delay care-recipient institutionalisation [1,13,14]. Day respite, which is the focus of this study, involves the care-recipient spending varied hours of care in a centre outside their home during daytime hours.

The strength of evidence regarding the benefits of respite are somewhat mixed but there is some evidence that it reduces carer burden and depression symptoms and increases feelings of well-being [1]. Carers receive a break, feel less hostile to the care recipient and use less negative coping strategies [15,16]. When people with dementia attended day respite Mason et al. [16] found that carers experienced benefits from the improved sleep patterns and decreased behavioural problems in the care-recipient.

Evidence about the benefits for care-recipients is less clear. Studies in North America did not record any significant effects for disabled elderly people attending day respite services [17,18]. An Irish study of younger disabled adults (average age 50 years) found that attendance at a day activity centre increased feelings of well-being but did not improve health [19], though whether these benefits apply to older adults attending day respite services is unknown. However, a study of 257 older persons attending social senior centres in USA, found that many (n = 173) felt their lives had improved as a result of attending the centre [20]. The major reasons for attending these centres were for friends and social support (n = 157) with meals (n = 35) and activities (n = 28) being less important. Logistic regression analysis showed that having friends at the centre lowered the risk of depressive symptoms in participants by 4-5%. While this study suggests useful research avenues, the participants were not attending a day respite service, limiting the messages that can be drawn from this study.

### Carer expectations, perceptions and trust

Encouraging and maintaining carer ongoing use of respite services can be an issue and the need for carers to trust service providers has been raised in several studies [3,21,22]. Logistic regression modelling of 113 carer survey responses found that negative beliefs about care-recipient outcomes was strongly associated with non-use of day respite for dementia care-recipients [12]. These authors remind us that the beliefs and attitudes of carers can have a strong influence on respite use. It seems likely that carers want to know that respite services are ‘good care’ and if care-recipient experiences do not meet carer expectations then carers may withdraw their commitment to using respite services.

Qualitative studies highlight the importance of emotional concerns in respite decisions where initial reluctance from care-recipients and carer guilt can lead to the abandonment of day respite [23,24]. Carers’ service choices are complex as carers attempt to balance their own needs with the needs of the care-recipient. Factors such as personality traits, financial costs and decisional conflict are all known to impact decisions [25-27]. A qualitative study of 18 rural carers of people with dementia found that carers felt great responsibility for the care of the care-recipient and carefully vetted health care providers before ceding responsibility for even short periods [3]. While in-home respite was well used, day respite was not, as it represented a greater ‘handing over of responsibility’ and a possible risk to the comfort and emotional well-being of the care-recipient [3 p.13]. These authors argue that carer support needs cannot be understood in isolation from the carer/care-recipient relationship.

There has, however, been little research conducted on understanding why carers use day respite services, including the respite service practices that influence care-recipient experiences and whether these match carer expectations. This study addresses this gap and explores carers’ a) expectations and experiences of day respite, and b) the levels of trust in care-recipients’ care.

## Methods

A mixed-method (i.e. the use of both quantitative and qualitative data) case study approach was used to answer the research questions. Yin [28] states that case studies investigate a phenomenon in their real-life context, and Kitchener [29] highlights the paradigmatic suitability of mixed methods case studies. A case study method was chosen because the researchers needed to relate carers’ responses to a deep knowledge of the type of service provided. The study ran from July to December 2011. Ethics approval was granted for the study by the Tasmanian Social Science Human Ethics Committee.

Psychological contract theory provided a useful framework to link the study elements of service provision, carer expectations, experiences, perceptions of care-recipient benefits and trust in the service. According to the theory, there are two aspects to psychological contracts: transactional contracts that focus on monetary exchanges and relational contracts that focus on social and emotional concerns [30]. While the theory is typically applied in employment studies its recent application in the health sector and the informal workforce of volunteers has proved insightful [31,32]. Psychological contract focuses on understanding mutual expectations between organisations and employees, or in this case, clients and it was used to explore whether carers’ perceptions and expectations of the day respite service are being met [33]. Whether carers feel the psychological contract is fulfilled or breached will impact on their ongoing use of respite services.

### Study setting

We recruited participants from our case study using a nested sampling strategy [28] where all current day respite service users formed the study population. The day respite Centre was a Southern Tasmanian adult day respite service situated within an aged care facility and is open six days per week for 12 hours per day. The service was initially funded in 2007 as a ‘demonstration site’, aiming to provide maximum flexibility for carers, and had aimed to provide a ‘club like’ atmosphere for care-recipients who are called ‘guests’. In the 12 month period concurrent with the study, services were provided to 126 care-recipients, with 93% being over aged 65 and those younger having age related illnesses such as early onset dementia and dementia associated with other diseases such as Huntington’s and Parkinson’s Disease. Of the 126 care-recipients, 26% had a disability, 36% had dementia, 16% had dementia with challenging behaviours, and 9% were identified as from culturally and linguistically diverse populations.

### Participant recruitment

All primary carers (as identified in service documentation) of users of the day respite service (126) were invited to participate via mailed letter and newsletter articles with 50 recruited to the study, a 40% response rate. The letter/newsletter provided contact details for one of the facility’s staff. This staff member provided an expression of interest form and an information sheet to interested carers, who could then either verbally inform the staff member of their interest or return the form. Researchers then contacted interested carers and gained verbal informed consent. Carers were then interviewed at a time and location that suited them; in some cases interviews were conducted by telephone.

### Data collection and analysis

The interview/survey was developed to elucidate carer expectations and perceived benefits of day respite services. Carers were asked to assess the outcomes of day respite on care-recipients, with items focused on quality of life and physical and emotional well-being. There are no established tools available which address carers’ perception of respite benefits for care-recipients. The survey items [see Additional file 1 – Respite Survey] were based on current literature and previous qualitative work undertaken by the authors with questions targeting mood, sociability, physical activity, physical health, activities of daily living (ADL) and instrumental ADL adapted from existing tools [34-37]. Carers were asked to rate items on care-recipients’ behaviour and well-being since attending day respite based on a three-point scale of more, the same or less. A group of questions also specifically focused on carers’ expectations of respite and the significance of trust in service provision. The interview was structured to provide sufficient open-ended questions to allow carers to voice their opinions. The interviewer-administered questionnaire took approximately 30 minutes. The interviewer was a doctoral candidate with interview experience. This approach provided mixed-method data, and ensured data was collected in a personal manner that helped to engage the often-elderly participants.

Quantitative data was descriptively analysed using SPSS Version 18 and key variables were cross-tabulated. Qualitative data underwent content analysis, assessing for recurring themes; these were then discussed amongst the research team. The analysis integrated organisational level knowledge with the interview survey results and researchers considered how the descriptions from all data informed the concepts of interest – expectations, perceived benefits, and trust looking for commonality and diversity [28]. The initial descriptive themes were emotional gains, social gains and meaningful activity, however researchers noted how well these mapped against Maslow’s theory of hierarchy of needs [38] and the utility of this approach in understanding quality of life (QoL) in dementia [35]. Levels one, two, three, and four of Maslow’s hierarchy of needs which are biological and physiological needs, safety needs, belongingness and love needs, and esteem needs respectively, were incorporated into the final themes. This approach follows a ‘best fit’ framework analysis [39] that allows the identification of further themes when *a priori* framework proves inadequate for explaining the data. The qualitiative component of the study adheres to the RATS guidelines [40].

## Results

The findings are explained under the three key themes ‘Carer Expectations – Four levels of needs from Maslow’s hierarchy’, ‘changes noticed since commencing day respite’, and ‘staff and trust – and important parameter for carers’. Our sample of carers were highly satisfied and committed to ongoing use of the service. Care-recipient enjoyment of day respite was central to carers’ ongoing commitment to using the service.

The mean age of respondents was 60 years (range 38–86) and the length of time providing care was variable amongst respondents and ranged from 6 months to 20 years, with an average time of almost 6 years. Data collection methods meant that researchers could not analyse the characteristics of the 60% non-respondents. However, the sample of carers recruited to the study had characteristics consistent with carers in Australia [2,4], in terms of sex (70% female), relationship to care recipient (42% spouse and 36% child) and age, suggesting a reasonable spread of respondents, (though Australian data has limitations [10]). Other respondent characteristics of note were; living situation (60% lived with the care recipient), employment (66% were not formally employed, 16% worked full-time, 14% worked part-time hours), and use of external service support (70% were receiving help from other services).

Through carer reports the average age of care-recipients was 78 (Range 60–95); 50% were female and 50% were male. On average (5% trimmed median) attendees spent 7.9 hours (range 1–45) at day respite per week and had been attending it for an average of 21 months (range 1–72). Table 1.

**Table 1:** Participant characteristics

		**Carer *Mean (Range) *** **count (%)**	**Care recipient *Mean (Range) *** **count (%)**
**Age**		*60 (38–86)*	*78 (60–95)*
**Sex**	Male	15 (30)	25 (50)
	Female	35 (70)	25 (50)
**Relationship to care recipient**	Husband	5 (10)	
	Wife	16 (32)	
	Son	6 (12)	
	Daughter	12 (24)	
	Other relative	8 (16)	
	Friend	3 (6)	
**Living with care recipient**	Yes	30 (60)	
	No	20 (40)	
**Employment status**	Full-time	8 (16)	
	Part-time	7 (14)	
	Casual	2 (4)	
	Not working	33 (66)	
**Assistance received from other services**	Yes	35 (70)	
	No	15 (30)	

### Carer expectations – four levels of needs from maslow’s hierarchy

While carers expected lower level needs of Maslow’s hierarchy to be met, they perceived the benefits of day respite for care-recipients to be largely social and emotional, which are higher level needs. Carers were asked open-ended questions about what they wanted from the service and what their expectations of it were. Carers’ wants and expected gains were focused largely on benefits for the care-recipient. They wanted the care-recipient to have both lower level needs (biological, physical, and safety), and the higher level needs met through the use of day respite services. Carers clearly wanted good care (43 responses) demonstrated by responses such as *‘a caring and understanding environment’ (husband), ‘that she is well taken care of’ (son)* and ‘*a safe environment’ (wife).* But carers also wanted the care-recipient to have an enjoyable experience (33 responses) demonstrated by responses such as ‘*fulfilment because he has done something constructive with his time’ (wife)* and *‘enjoyment and some stimulation’ (daughter)*. Only 17 responses to this question were related to the carer’s needs, and of these 11 were related to the carer’s concern for the care-recipient with comments such as ‘*peace of mind that she is not alone (son)*. The comment *‘I hope he will enjoy himself sufficiently that he would stay there and allow me to do the things I can’t do with him in tow’ (wife)* demonstrates the connection carers feel between the care-recipient experience and use of day respite. There were only six responses directly related to carers’ interests: one ‘*wanted a break’*, one indicating that carers wanted the service to be cheaper, three wanted the service open for longer hours, and one saw the service as a ‘*stepping stone*’ to institutional care.

Carers felt that the care-recipients’ expectations and benefits were largely about emotional gains (18 responses), social gains (14 responses) and additional stimulation (8 responses), with these elements often combined in the one response. For example: ‘*the interaction with other men, the Men’s shed and feeling useful’ (daughter)* and *‘she loves the company, she loves the staff, she would love to win the bingo, she loves getting out and the activities’ (daughter).* These responses are all situated within levels three and four of Maslow’s hierarchy; -belongingness, love and esteem needs. Carers’ expectations are focused on the care-recipient’s sense of enjoyment and feelings of fulfilment on attending the centre with an increase in their overall quality of life. Carers expected that care-recipients would make friends and have social interactions with others outside of the home while undertaking interesting activities that would help keep them more physically and mentally active.

Almost all respondents indicated that from a choice of mostly, sometimes or never, their expectations were largely met by day respite care. These themes also emerged from responses to questions about the perceived benefits and most enjoyed elements of day respite for care-recipients. Socialising (36 responses), activities (32 responses), and outings (18 responses) were the key themes from the content analysis, which are also consistent with those higher level needs.

The belongingness and love benefits reported by carers included companionship and social interaction, with some care-recipients reported to have developed close friendships, for example, *‘she likes catching up on the gossip and the girls…’ (friend)*. Attendees not only enjoyed interactions with their peers but they also enjoyed interactions with the staff; *‘the interaction with people – staff and clients…’ (wife)*. Esteem benefits were found in the meaningful activities, with the centre catering to different gender needs such as the Men’s Shed. Activities also supported feelings of usefulness with value adding activities reflecting the ethos of the centre for example: *“It is a chance to continue his interest in woodwork and moulding”.* Care-recipients were often able to display their work and offer them for sale at the Centre. Some carers commented on how care-recipients felt *‘useful’ (daughter)* or ‘*said they treat me like a human-being*’ *(daughter).* Further, where appropriate, staff also facilitate care-recipients to actively participate in the centre by helping out, for example assisting in preparing refreshments. This carer reported *‘He thoroughly enjoys getting out and meeting people and enjoys the responsibility given to him’ (wife).*

### Changes noticed since attending day respite

Carers were asked to rate whether care-recipients exhibited a range of behaviours or emotions more often, the same or less often since attending day respite (see Table 2). The greatest benefits or improvements for the care-recipient were mostly associated with social and emotional gains rather than physical gains. Carers found care-recipients were more interested in friends and daily activities (66%), to be in good spirits most of the time (64%), and were more physically active (56%). While the social interaction between carer and care recipient was also reported to have increased (56%).

**Table 2:** Perceived benefits for care-recipients

**Since starting respite care does/is your relative:**	**More**	**Same**	**Less**
	**Count (%)**	**Count (%)**	**Count (%)**
Physically active within the limits of their age and illness?	28 (56)	16 (32)	6 (12)
Interested in friends and daily activities	33 (66)	15 (30)	2 (4)
In good spirits most of the time	32 (64)	18 (36)	0 (0)
Preferring to stay home rather than going out	11 (22)	26 (52)	13 (26)
Able to remember things	9 (18)	27 (54)	14 (28)
Worried about the future	3 (6)	38 (76)	9 (18)
Restless during the daytime	5 (10)	35 (70)	10 (20)
Restless during the night-time	1 (2)	46 (92)	2 (4)
Taking care of own personal hygiene	8 (16)	37 (74)	5 (10)
Helping with chores	8 (16)	29 (58)	13 (26)
Dressing without assistance	2 (4)	40 (80)	8 (16)
Continent	1 (2)	36 (72)	13 (26)
Having solid sleep	6 (12)	38(76)	5 (10)
Socially interactive with yourself or others	28 (56)	20 (40)	2 (4)

There were no major improvements reported in the care recipients’ physical behaviours, but decreases in the following parameters were identified; helping with chores (26%), continence (26%) and ability to remember things (28%). Some carers made the point that these decreases were due to the advancement in the care-recipient’s disease or advancing age rather than attributed to their attendance at day respite.

### Staff and trust - an important parameter for carers

To gain an understanding of the relational contract between the service and carers, a range of questions were asked about trust, communication and staff.

As outlined in Table 3 the majority of carers (90%) indicated that their relative’s enjoyment has ‘a lot’ of influence on their commitment to care-recipients attending day respite services.

**Table 3:** Carers responses to questions about trust in respite

	**A lot**	**Somewhat**	**Not at all**
	**Count (%)**	**Count (%)**	**Count (%)**
How much does your relative’s enjoyment influence your commitment to them coming to the centre in the future?	45 (92)	4 (8)	0 (0)
Do you ever feel guilty that your relative comes to the centre?	0 (0)	6 (12)	43 (88)
Is care of your relative primarily your responsibility?	38 (78)	9 (18)	2 (4)
How well do you feel you know the staff at the centre?	14 (29)	24 (49)	11 (22)
How much trust do you have in the centre’s care?	46 (94)	3 (6)	0 (0)
Do you think your relative is in ‘safe hands’?	48 (98)	1 (2)	0 (0)
Do you feel the staff are kind?	47 (96)	1 (2)	0 (0)
Do you think the staff know about your relatives/spouse/friend health and family circumstance?	30 (61)	13 (27)	2 (4)
Do you feel the staff listen carefully when you give them information concerning your relative?	36 (74)	7 (14)	1 (2)
Overall, do you enjoy the break?	40 (82)	3 (6)	1 (2)

Very high proportions also indicated that carers had ‘a lot’ of trust in the care provided (92%) and thought that their relative was in ‘safe hands’ (96%) and felt that staff ‘are kind’ (94%). Results from Table 2 below show that to achieve trust it is not always necessary for carers to know staff well, as less than half of the carers (48%) indicated that they felt they knew staff only ‘somewhat’ and 22% indicated ‘not at all’. Additionally two-thirds of carers did not feel that staff knew “a lot” about their relative’s family circumstances. It is possible that some carers rely on feedback about staff and care from care-recipients and can therefore feel trust in the staff without needing to know the staff themselves.

While the results highlight that care-recipients’ enjoyment/satisfaction with respite is very important for carers’ commitment to using services, 80% of carers also appreciate the opportunity for a break from caring ‘a lot’. Only 12% felt somewhat guilty that their relative attended respite, and this may relateto the care-recipient’s enjoyment in attending the Centre and the trust carers have in the services provided.

Carers were asked open-ended questions about what they liked most about the service. Following content analysis the greatest response categories were related to: staff (32 responses) - *‘staff very caring’ (other relative)*, activities (14 responses) - *good program of activities (daughter),* and carer respite (14 responses) - *gives me a break and I know he’s not home alone and is safe (wife)*. Some carers linked the service to their ability to provide ongoing care, or to continue in paid employment. *‘Because they provide a good day service, I am able to keep her at home’*.

The largest response category when asking carers what they liked about the service was related to the staff of the day respite service (32 responses). Words such as caring, friendly, attentive, patient and professional were frequently used to describe staff. The few negative comments also highlight the importance of staff having good skills and attitudes towards care-recipients with two comments about one staff member who was ‘*patronizing’* and ‘*treats them like children’*.

## Discussion

Both Maslow’s hierarchy of needs and psychological contract theory have proved useful frameworks for considering why carers use day respite services. Carers expect quality care that provides for care-recipients’ higher level needs. Carers need to trust these services since they relinquish their control over the care of the care-recipient for a given time period. To trust the service they must believe that services will meet their expectations – primarily that care-recipients will receive benefits from attending day respite. The study has also highlighted that carers perceive care-recipients benefits are largely social and emotional, not physical. These results are consistent with other reported research [19,20]. The levels of trust and perceived benefits described by carers explain why they do not feel guilty in using the service, unlike carers of people with dementia reported in Orpin et al. [3].

Using the first four levels of Maslow’s hierarchy of needs [38], we can see that carers’ expect higher-level benefits for care-recipients, in addition to the first two levels of physiological care and safety. Carers also expect day respite to meet care-recipient needs at the third level, for love and belongingness, through friendship and social acceptance. Additionally, carers expect that day respite services will meet care-recipients self-esteem needs through respectful treatment and meaningful activity. These findings expand on the work of Phillipson, Magee and Jones [12] by highlighting that care-recipients’ enjoyment of day respite is central to carers ongoing commitment to using the service, and this allows them to enjoy the break without guilt. They also further Orpin, Stirling, Hetherington & Robinson’s [3] contention that the carers’ feelings of responsibility for the care-recipient led to vigilance in selecting services with a focus on standards of care and care-recipient well-being.

This study has highlighted the usefulness of Maslow’s hierarchy in understanding expectations of quality care when carers’ expectations can be mapped so closely to the hierarchy. Scholzel-Dorenbos, Meeuwsen, and Rikkert [35] have demonstrated how well Maslow’s hierarchy maps against QoL measures in understanding care for people with dementia. Other work supports the importance of these higher level needs, [40] finding that the benefits of day centres are largely due to the social and emotional impacts on care-recipient’s well-being [19,20]. We argue that this growing body of evidence suggests services can use Maslow’s hierarchy of needs to consider service re-design so that services meet higher levels needs than safety and physiological care.

### Limitations

This is a single case study of carers from one respite service centre precluding any definitive conclusion or generalizations; additional studies examining carers’ expectations of day respite services are required. The responses were strongly positive but this could be related to the 40% response rate as only the most concerned or engaged carers may have responded or the use of service providers to assist in recruitment may have biased recruitment towards carers with more positive views of the service. We suggest future research could use a multi case study approach, seeking participant carers and care-recipients from mulitple day respite services.

## Conclusions

In summary, this exploratory case study has supported recent work by others suggesting that carers need to feel confident that day respite services provide high level benefits to care-recipients that include belongingness, love and esteem if carers are to take up the benefits of a ‘respite break’. Trust in quality care that includes social interactions along with meaningful and enjoyable activities for care-recipients are central issues for carers who use day respite. Maslow’s hierarchy of needs proved most useful for understanding carers’ expectations of respite services.

## Competing interests

This study was funded by Glenview Community Services and reports on their services, but they did not have any influence on data analysis or reporting.

## Authors’ contributions

CS contributed to the study design, data analysis, and writing of the paper. CD contributed to data collection, data analysis, and writing of the paper. AM contributed to the study design and writing of the paper. All authors read and approved the final manuscript.

## Pre-publication history

The pre-publication history for this paper can be accessed here:

http://www.biomedcentral.com/1472-6963/14/245/prepub

## Supplementary Material

Additional file 1 Adult day care respite: caregiver expectations and benefits survey. Click here for file
