# Supplementary material for: Why carers use adult day respite: a mixed method case study
Source: BMC Health Serv Res. 2014 Jun 6;14:245. doi: 10.1186/1472-6963-14-245 (PMC4068069; doi:10.1186/1472-6963-14-245)
Supplement: Additional file 1 — Adult day care respite: caregiver expectations and benefits survey. [file 1472-6963-14-245-S1.pdf]

Section 1 Caregiver details – circle correct response

Age	
Gender	Male Female
Relationship to care recipient	Husband Wife Son Daughter Other relative Friend
Living with care recipient?	Yes No
Length of time providing care for care recipient?	
Employment status – i.e	Full time Part time Casual Not formally employed

Section 2 Care recipient details – write in full

Age	
Gender	
Is assistance received from other services?	

Section 3 We are interested in how attending Bidsdee influences the health and well-being of your relative/spouse/friend. Can you tell me whether you feel attending Bisdee has meant your relative has more, the same or less of the characteristics in the following items:

	**Activities and Functions**			
**1**	Physically active within the limits of their age and illness	*More*	*Same*	*Less*
**2**	Interested in friends and daily activities	*More*	*Same*	*Less*
**3**	In good spirits most of the time	*More*	*Same*	*Less*
**4**	Prefers to stay home rather than going out	*More*	*Same*	*Less*
**5**	Able to remember things	*More*	*Same*	*Less*
**6**	Worried about the future	*More*	*Same*	*Less*
**7**	Restless during the daytime	*More*	*Same*	*Less*
**8**	Restless during the night-time	*More*	*Same*	*Less*
**9**	Taking care of own personal hygiene	*More*	*Same*	*Less*

**10**	Helping with chores	*More*	*Same*	*Less*
**11**	Dressing without assistance	*More*	*Same*	*Less*
**12**	Continent	*More*	*Same*	*Less*
**13.**	Solid sleep	*More*	*Same*	*Less*
14.	Social interaction with yourself or others	*More*	*Same*	*Less*

Why does your relative come to Bisdee? ……..

Do you think your relative understands why they come to Bisdee? Yes/No

Does your relative like coming to Bisdee? Yes/No

How many hours a week do they spend here? …………..

How long have they been coming? ……… weeks/months/years

Have they previously attended day respite facilities, other than Bisdee?   Yes/No   If Yes why change?...

What would you say are the things he/she most enjoys about Bisdee? ……………………………………..

……

What do you hope the person you care for would get out of coming to Bisdee?   Are your hopes met?:

………   Mostly   Sometimes   Never

……   Mostly   Sometimes   Never

………   Mostly   Sometimes   Never

What things do you most like about Bisdee? ………………………………………………………………………………………………

What do you expect from Bisdee service? Are your expectations met: Mostly Sometimes Never

……………………………………………………………………………………………………… Mostly Sometimes Never

………. Mostly Sometimes Never

…….. Mostly Sometimes Never

Do you feel the care here is the same as 'at home'? Yes/No? How is it the same or different? ………………………………………………………………………………..

………

For the following questions can you tell us whether you feel the answer is a lot, somewhat, or not at all?

**1**	How much does your relative's enjoyment influence your commitment to them coming to Bisdee in the future?	*A lot*	*Somewhat*	*Not at all*
**2**	Do you ever feel guilty that your relative comes to Bisdee?	*A lot*	*Somewhat*	*Not at all*
**3**	Is care of your relative primarily your responsibility?	*A lot*	*Somewhat*	*Not at all*
**4**	How well do you feel you know the staff at Bisdee?	*A lot*	*Somewhat*	*Not at all*
**5**	How much trust do you have in Bisdee care?	*A lot*	*Somewhat*	*Not at all*
**6**	Do you think your relative is in 'safe hands' here?	*A lot*	*Somewhat*	*Not at all*
**7**	Do you feel the staff here are kind?	*A lot*	*Somewhat*	*Not at all*
**8**	Do you think the staff here know about your relatives/spouse/friend health and family circumstances?	*A lot*	*Somewhat*	*Not at all*
**9**	Do you feel the staff here listen carefully when you give them information concerning your relative?	*A lot*	*Somewhat*	*Not at all*
**10**	Overall, do you enjoy the break	*A lot*	*Somewhat*	*Not at all*

How would you describe Bisdee to another carer? …….

………..

Do you have any advice for Bisdee?..
